# Triggering Receptor Expressed on Myeloid Cells-2 (TREM2) Interacts With Colony-Stimulating Factor 1 Receptor (CSF1R) but Is Not Necessary for CSF1/CSF1R-Mediated Microglial Survival

**DOI:** 10.3389/fimmu.2021.633796

**Published:** 2021-03-25

**Authors:** Baoying Cheng, Xin Li, Kai Dai, Shengshun Duan, Zhouyi Rong, Yingmin Chen, Liangcheng Lü, Zhaoji Liu, Xiaohua Huang, Huaxi Xu, Yun-Wu Zhang, Honghua Zheng

**Affiliations:** ^1^Fujian Provincial Key Laboratory of Neurodegenerative Disease and Aging Research, Institute of Neuroscience, School of Medicine, Xiamen University, Xiamen, China; ^2^Basic Medical Sciences, School of Medicine, Xiamen University, Xiamen, China

**Keywords:** TREM2, CSF1R, interaction, cell survival, microglia

## Abstract

Triggering receptor expressed on myeloid cells-2 (TREM2) and colony-stimulating factor 1 receptor (CSF1R) are crucial molecules for microgliopathy, which is characterized by microglia dysfunction and has recently been proposed as the neuropathological hallmark of neurological disorders. TREM2 and CSF1R are receptors expressed primarily in microglia in the brain and modulate microglial activation and survival. They are thought to be in close physical proximity. However, whether there is a direct interaction between these receptors remains elusive. Moreover, the physiological role and mechanism of the interaction of TREM2 and CSF1R remain to be determined. Here, we found that TREM2 interacted with CSF1R based on a co-immunoprecipitation assay. Additionally, we found that CSF1R knockdown significantly reduced the survival of primary microglia and increased the *Trem2* mRNA level. In contrast, CSF1R expression was increased in *Trem2*-deficient microglia. Interestingly, administration of CSF1, the ligand of CSF1R, partially restored the survival of *Trem2*-deficient microglia *in vitro* and *in vivo*. Furthermore, CSF1 ameliorated Aβ plaques deposition in *Trem2*^-/-^; 5XFAD mouse brain. These findings provide solid evidence that TREM2 and CSF1R have intrinsic abilities to form complexes and mutually modulate their expression. These findings also indicate the potential role of CSF1 in therapeutic intervention in TREM2 variant-bearing patients with a high risk of Alzheimer’s disease (AD).

## Introduction

Triggering receptor expressed on myeloid cells-2 (TREM2), an immunomodulatory receptor dominantly expressed in myeloid lineage cells such as dendritic cells, osteoclasts and microglia, is essential in activation and survival of myeloid cells. The genetic variants of TREM2 result in an increased risk of several neurodegenerative disorders, including Alzheimer’s disease (AD) (1–5). TREM2 consists of an extracellular V-type Ig domain followed by a short stalk (ectodomain = 19–172 aa), leading to a single transmembrane helix that interacts with DNAX-activation protein 12 (DAP12, also known as TYROBP) containing the immunoreceptor tyrosine-based activation motif (ITAM) to mediate downstream signaling (6, 7). We have previously demonstrated that TREM2 deficiency reduced the viability and proliferation of primary microglia, and induced cell cycle arrest at the G1/S checkpoint (8), indicating an important role of TREM2 in microglia survival.

Colony stimulating factor 1 receptor (CSF1R) is also predominantly expressed in monocyte, macrophage and bone marrow cell precursors, fetal trophoblast and chorioma cells, as well as microglia in the central nervous system (CNS) (9–11). Studies have revealed that people with CSF1R variants bear an adult-onset neurodegenerative disorder characterized by dementia and motor impairments, named adult-onset leukoencephalopathy with axonal spheroids and pigmented glia (ALSP) (12, 13). Thus, TREM2 and CSF1R are considered crucial molecules for “microgliopathy”, which is characterized by reactive gliosis and microglia dysfunction and has been recently proposed as the neuropathological hallmark of neurological disorders (5).

The activation of CSF1R by its ligand colony-stimulating factor 1 (CSF1), also known as macrophage CSF (M-CSF), regulates the survival, proliferation and chemotaxis of macrophages and osteoclasts (14). TREM2 is involved in CSF1-mediated survival and proliferation in macrophages and osteoclast precursors and CSF1 signals through CSF1R to activate β-catenin in a TREM2-dependent manner in those cells (15, 16). In *Trem2*^-/-^ mice, the β-catenin deficit in osteoclast precursors reduces proliferation and prematurely accelerates osteoclast maturation (15). Another study showed that CSF1 stimulation resulted in DAP12 phosphorylation by Src kinases activated by CSF1R in osteoclasts (17). These results suggest that TREM2 and CSF1R are in close physical proximity (6). However, whether there is a direct interaction and regulation between these receptors remains unknown. Moreover, given the crucial role of TREM2 and CSF1R in microglia survival, the precise mechanisms by which these receptors regulate microglia survival and function still await full exploration.

In the current study, we examined the interaction of CSF1R and TREM2 using transient transfection of these two proteins in HEK293T cells. We demonstrated that TREM2 interacted with CSF1R. Moreover, CSF1R was significantly increased in *Trem2*^-/-^ microglia whereas downregulation of CSF1R impaired the survival of microglia and increased the *Trem2* mRNA level. Interestingly, activating CSF1R by CSF1 resulted in significant activation of the Akt signaling pathway, and partially restored the attenuated cell survival in *Trem2*^-/-^ microglia and in *Trem2*^-/-^ mouse brain. Furthermore, administration of CSF1 significantly ameliorates Aβ plaques accumulation in *Trem2*^-/-^; 5XFAD mouse brain. Thus, our results indicate that TREM2 interacts with CSF1R but is not necessary for CSF1R-mediated microglia survival. This study also provides a viable target for therapeutic intervention by enhancing CSF1R function in TREM2-loss-of-function patients.

## Materials and Methods

### Reagents

Phospho-CSF1R (Tyr723) (49C10) Rabbit mAb (RRID: AB_2085229), β-catenin (6B3) Rabbit mAb (RRID: AB_331149), anti-Phospho-Akt (Ser473) (RRID: AB_329825) and anti-total-Akt antibodies (RRID: AB_329827) were all bought from Cell Signaling Technology. Anti-GAPDH (RRID: AB_2630358), anti-CSF1R (RRID: AB_2085251), anti-Aβ (MOAB2), anti-β-actin antibodies (RRID: AB_306371) and Alexa Fluor 647 Donkey Anti-Rat IgG H&L (RRID: AB_2813835), as well as the BrdU Cell proliferation ELISA Kit, were all from Abcam. Rat Anti-mouse CD68 monoclonal antibody (RRID: AB_322219) was from Bio-Rad. Anti-HA-tag (RRID: AB_11042321) antibody and anti-Myc-tag antibody (RRID: AB_11182162) were from Proteintech. Rabbit anti-Iba1 (RRID: AB_2687911) was from Wako. Alexa Fluor 488 Goat anti-Rabbit (RRID: AB_10374301), Alexa Fluor 594 Goat anti-Rabbit (RRID: AB_10374440), Alexa Fluor 647 Goat anti-Rabbit antibodies (RRIDAB_10371940) and TRIzol reagent were all from Thermo Fisher Scientific. Granulocyte-macrophage colony stimulating factor (GM-CSF) and CSF1 were from R&D Systems. Basic Glial Cells Nucleofector Kit was purchased from LONZA. ReverTra Ace qPCR RT Master Mix was from TOYOBO. FastStart Universal SYBR Green Master mix was from Roche. CellTiter 96^R^ Aqueous One Solution (MTS assay) and DeadEnd™ Fluorometric TUNNEL System were from Promega.

### Culture and Treatment of Primary Microglia

Primary microglial cells were prepared as described previously (8). Briefly, mixed glial cells from newborn (postnatal 1 or 2 d) *Trem2^-/-^* or WT C57BL/6 pups were cultured in Dulbecco modified Eagle’s medium (DMEM) supplemented with 10% fetal bovine serum (FBS) and 100 U/ml penicillin/streptomycin in poly-L-lysine (Sigma-Aldrich)-coated cell culture flasks (Thermo). The media was replaced the next day with fresh DMEM plus 10% FBS and 25 ng/ml GM-CSF. Microglia cells were harvested by shaking at a speed of 200 rpm for 20 min after 10 days in culture. The isolated WT microglial cells were subjected to CSF1R knockdown by electroporation. Isolated microglia from *Trem2^-/-^* or WT littermates were plated for MTS assay, BrdU incorporation assay, or terminal deoxynucleotidyl transferase-mediated-deoxyuridine triphosphate nick-end labeling (TUNEL) staining. For rescue experiments, *Trem2^-/-^* or WT microglia were treated with 50 ng/mL CSF1 for 24 hours and used for qRT-PCR, Western blotting, MTS assay, BrdU incorporation assay, or TUNEL staining.

### Animals and Brain Stereotaxic Injection

#### Short-Term Injection of CSF1 in *Trem2*^-/-^ Mouse Brain

TREM2 knockout (KO) mice (*Trem2^-/-^*; C57BL/6) and wild type (WT) C57BL/6 littermates were obtained from the UC Davis Knockout Mouse Project (KOMP) repository as described previously (18) and were group housed (12 hr/12 hr light/dark) in the Laboratory Animal Center of Xiamen University. Brain stereotaxic injection was performed as described previously (19). Briefly, two-month-old *Trem2^-/-^* and age matched WT mice were anesthetized and placed in a stereotaxic frame. A skin incision was made, and holes were drilled at x (1.0 mm from bregma) and y (−0.5 mm from bregma). Sterile PBS (3.0 μL) with or without 30 ng CSF1 was delivered into the right cerebral ventricle at 0.15 µL/min at z-depths of 2.3 mm. The syringe was left in place for 10 min after each injection before being withdrawn slowly. Following injection daily for three days, mice were anesthetized, and the brains were dissected. For biochemical analysis, hippocampus and cortex from the left brain were dissected out, snap-frozen in liquid nitrogen, and stored at −80 °C for later extraction of protein and RNA. For histologic analysis, the right hemisphere was fixed with 4% PFA overnight at 4 °C and transferred to 30% sucrose for 48 hours before being embedded for cryostat sectioning.

#### Long-Term Injection of CSF1 in *Trem2*^-/-^; 5XFAD Mouse Brain

Long-term injection of CSF1 was carried out according to our previous study (20). Briefly, male *Trem2*^-/-^; 5XFAD mice (3 to 3.5 months of age) were anaesthetized and stereotaxically injected with 3 μl of 10 ng/μL CSF1 into the right lateral ventricle per week (0.5 mm lateral to the midline, 1.0 mm posterior to the bregma, at a depth of 2.3 mm). Mice were anaesthetized and perfused 2.5 months later. Vehicles were used as Artificial cerebrospinal fluid (ACSF) - treated controls in these experiments. Brains were obtained and post-fixed overnight at 4°C in 4% PFA in PBS. Coronal sections from mouse brains were subjected to Iba1 (1:400), CD68(1:200) and MOAB2 (1:400) immunofluorescence staining and were assessed by independent observer blind to animal treatment groups.

### Co-Immunoprecipitation Assay

HEK293T cells were co-transfected with HA-tagged CSF1R and Myc-tagged TREM2. Twenty-four hours later, cells were harvested and lysed in 1% TNEN (50 mM Tris-HCl pH 8.0, 150 mM NaCl, 2 mM EDTA, 1% NP-40) lysis buffer supplemented with protease inhibitors. For all experiments, total protein concentration was determined by bicinchoninic acid (BCA) assay (Boster). Lysates were immunoprecipitated using anti-Myc or HA antibodies in the presence of Protein G Dynabeads (Thermo Fisher), followed by immunoblot analysis.

### CSF1R Knockdown by siRNA

Electroporation in primary microglia was performed as described by us previously (8). Briefly, knockdown of CSF1R with *Csf1r*-specific siRNA (GenePharma) was achieved by electroporation using an Amaxa Nucleofector and a glial specific Nucleofector kit (LONZA) according to the manufacturer’s instructions. Each electroporation reaction contained 10^6^ cells and 200 nM siRNA. Transfected cells were plated and used for qRT-PCR, Western blotting, MTS assay.

### TUNEL Staining

Cells were fixed in 4% polyformaldehyde (Sigma-Aldrich) for 15 min, and then subjected to TUNEL staining according to the manufacturer’s instructions (Dead End Fluorometric TUNNEL System, promega, G3250). DAPI was used to stain cell nuclei. Seven different fields per HPF were selected randomly to quantify the number of TUNEL-positive cells. TUNEL-positive cell nuclei were counted manually and compared with the total microglia number counted manually as DAPI and given as a percentage. The quantification data were from at least three independent experiments.

### Reverse Transcription and Quantitative Real-Time PCR

Total RNA was isolated from mouse brain or primary microglia using TRIzol reagent (Thermo Fisher Scientific). Reverse transcription was performed using ReverTra Ace qPCR RT Master Mix (TOYOBO) and quantitative PCR (qPCR) was performed using the FastStart Universal SYBR Green Master mix (Roche). The set of β-actin primers was used as an internal control for each specific gene amplification. The qPCR was performed on a LightCycler 480 (Roche). The real-time value for each sample was averaged and compared using the cycle threshold (CT) method, where the amount of target RNA (△CT) was normalized to the endogenous β-actin reference (CT) and then normalized against control levels. The primer sequences were as follows: *β-actin*-forward: 5’-TCTTGGGTATGGAATCCTGTGGCA-3’; *β-actin*-reverse: 5’-TCTCCTTCTGCATCCTGTCAGCAA-3’; *Csf1r*-forward: 5’-GGTTGTAGAGCCGGGTGAAA-3’; *Csf1r*-reverse: 5’-AAGAGTGGGCCGGATCTTTG-3’; *Trem2*-forward: 5’-TGCTGGCAAAGGAAAGGTG-3’; and *Trem2*-reverse: 5’-GTTGAGGGCTTGGGACAGG-3’.

### Western Blotting

Samples were homogenized and incubated in RIPA Lysis and Extraction Buffer (Boster), supplemented with Protease and Phosphatase Inhibitor Cocktail (Thermo). Protein concentrations were determined using the BCA Protein Assay Kit (Boster) according to the manufacturer’s instructions. Equal amounts of total proteins were resolved by sodium dodecyl sulfate–polyacrylamide gel electrophoresis and transferred to PVDF membranes (Millipore, IPVH00010). After blocking, the membranes were blotted using a primary antibody (1:1,000) and detected with horseradish peroxidase-conjugated secondary antibody (1:10,000). Proteins were visualized using ECL Western blotting detection reagents (Yeasen). Immunoreactive bands were quantified using ImageJ software (8).

### MTS Assay

Cell viability was analyzed using the MTS assay. Briefly, microglia were cultured in 96-well plates at a concentration of 2 × 10^4^/well and treated with or without 50 ng/ml CSF1. After 24 hours, 20 μl of MTS One Solution Reagent was pipetted into each well of the 96-well assay plate containing the cells in 100 μl culture medium. Then the plate was incubated at 37°C for 2 hours in a humidified, 5% CO_2_ atmosphere before recording the absorbance at 490 nm using an automated ELISA plate reader (Varioskan Flash, Thermo).

### BrdU Incorporation Assay

The BrdU incorporation assay was performed using the BrdU cell proliferation ELISA kit (Abcam) according to the manufacturer’s protocol. Briefly, microglial cells were seeded at a density of 10^4^ cells/well and BrdU was added to the cells for 24 hours for incorporation. Cells were then fixed, permeabilized, and denatured to enable the detection of incorporated BrdU through anti-BrdU antibody. After the incubation of horseradish peroxidase-conjugated secondary antibody, BrdU-positive ELISA signals were quantified using a spectrophotometer (Varioan Flash; Thermo Fisher Scientific) at a dual wavelength of 450/550 nm and normalized against total cell numbers.

### Immunofluorescence Staining

The coronal brain slices were fixed with 4% PFA in PBS for 30 min at room temperature (RT). Before staining, the slices were washed thoroughly with PBS. The slices were then permeabilized with 0.1% Triton X-100 for 15 min at RT. The nonspecific binding sites were blocked with 5% normal goat serum in PBS for 30 min at RT and the slices were incubated with the mouse anti-Iba1 primary antibody (1:400; Wako Chemicals) and/or MOAB2 (1:400) and/or CD68 (1:200) overnight at 4°C. The slices were then washed three times with PBS and incubated with the Alexa Fluor 488-conjugated goat anti-mouse secondary antibody (1:500; Thermo) for 2 hours at RT. The slices were washed three times with PBS and sealed with an anti-fade reagent (Life Technologies, P36935). The images were captured with an Olympus FV10-ASW confocal microscope. Five different fields per high-power field (HPF) in hippocampi and cortices of at least five sections per mouse from three to five mice were randomly selected to quantify the number of microglia double stained with Iba1 and/or MOAB2 with Olympus FV10-ASW 4.0 Viewer software.

### Statistics

Data were presented as mean ± SEM. Two groups were compared using two-tailed Student’s *t*-tests. For more than two groups, one- or two-way ANOVA was used, as appropriate, followed by Dunnett’s or Tukey’s *post hoc* adjustment. All analyses were performed with GraphPad Prism 7 software. *p* < 0.05 was considered statistically significant.

## Results

### TREM2 Interacts With CSF1R

**Figure 1:**
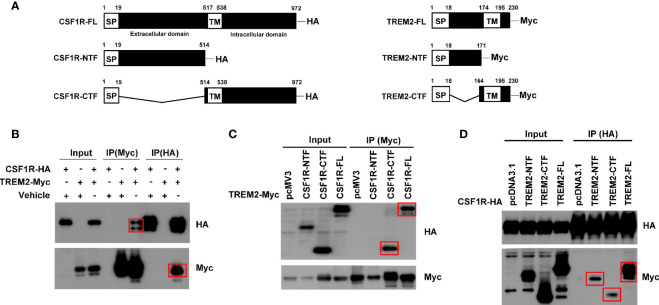
TREM2 interacts with CSF1R. HEK293T cells were transfected with different combinations of indicated plasmids, and Western blotting was performed using HA and Myc-tag antibodies. **(A)** Schematic diagram of fragments of CSF1R and TREM2. **(B)** Co-IP of HA-tagged full-length CSF1R with Myc-tagged full-length TREM2. **(C)** Co-IP of HA-tagged CSF1R fragments with Myc-tagged full-length TREM2. **(D)** Co-IP of HA-tagged full-length CSF1R with Myc-tagged TREM2 fragments. Red rectangles indicate positive bands.

It has been proposed that TREM2 and CSF1R are in close physical proximity (6). We thus explored their potential interactions by immunoprecipitation assays. We first generated full-length TREM2 or CSF1R plasmid and found that full-length TREM2 interacted with full-length CSF1R (**Figures 1A, B**). We then generated various TREM2 or CSF1R truncated constructs that respectively carry an additional Myc tag or HA tag at the carboxyl terminus (**Figure 1A**) to find the specific fragment of their interaction. We found that full-length TREM2 did not interact with the CSF1R amino-terminal fragment (NTF, 1 aa - 514 aa) (**Figure 1C**) but interacted with the CSF1R carboxyl-terminal fragment (CTF, 514 aa - 972 aa) (**Figure 1C**), which possesses the transmembrane domain. Reciprocally, full-length CSF1R also interacted with TREM2-CTF (164 aa - 230 aa) that contains the transmembrane domain (**Figure 1D**), and TREM2-NTF (1 aa -171 aa) that has no transmembrane domain (**Figure 1D**), indicating that the transmembrane of TREM2 and CSF1R are colocalized and interact with each other. Taken together, these results indicate that these two receptors can interact with one another.

### Increased Expression of CSF1R in *Trem2*^-/-^ Microglia

**Figure 2:**
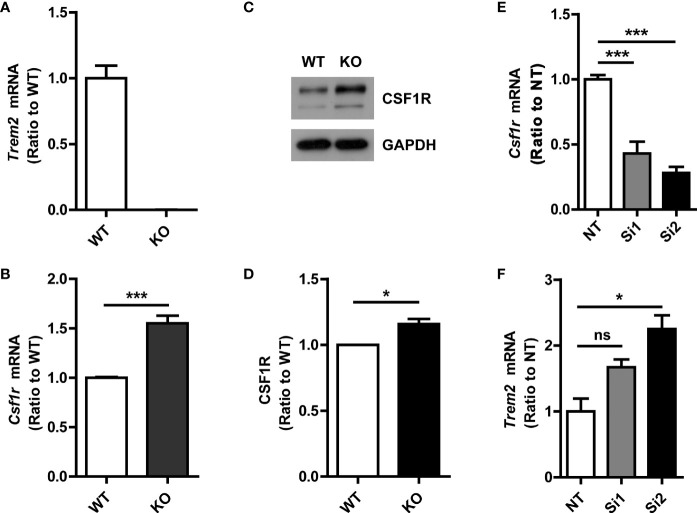
Increased CSF1R expression in *Trem2^-/-^* (knockout, KO) microglia. **(A)** *Trem2* KO in mouse primary microglia was confirmed by qRT-PCR. **(B)** *Csf1r* mRNA increased in *Trem2* KO primary microglia. **(C)** Representative images of Western blotting. **(D)** CSF1R was increased in *Trem2* KO primary microglia. Protein levels of CSF1R were quantified by densitometry and are presented as ratios to GAPDH. **(E)** *Csf1r* was knocked down in primary microglia by two independent siRNAs and the knockdown efficiency was assessed by qRT-PCR. **(F)** *Trem2* mRNA was increased in *Csf1r* knockdown primary microglia. One-way ANOVA with Dunnett’s multiple comparison test. Data are plotted as mean ± SEM (n=3). *p<0.05; ***p<0.001; ns, not significant.

Protein interactions mediate many types of cellular processes. To further explore the potential mutual effects of CSF1R and TREM2 on their expression, we determined the effect of TREM2 on CSF1R expression in primary microglia. The mRNA and protein levels of CSF1R in primary microglia from *Trem2^-/-^* or WT mice were detected by qPCR and Western blotting assay, respectively (**Figures 2A–D**). As expected, *Csf1r* mRNA was significantly increased in *Trem2^-/-^* microglia when compared to that in WT cells (**Figure 2B**). Similarly, CSF1R protein level was also enhanced in *Trem2^-/-^* microglia compared with WT cells (**Figures 2C, D**). Conversely, to further explore the effects of CSF1R on TREM2 expression, two independent *siRNAs* (Si1 and Si2) were used to knock down the expression of *Csf1r* in microglia and the knockdown efficacy was verified by qRT-PCR (**Figure 2E**). Compared to non-targeting (NT) control, *Trem2* mRNA transfected with *Csf1r* siRNAs was significantly increased (**Figure 2F**). Collectively, these data demonstrated that CSF1R and TREM2 mutually modulate their expression and the expression of CSF1R is increased in *Trem2* KO microglia.

### CSF1R Knockdown Significantly Decreases Microglial Survival

**Figure 3:**
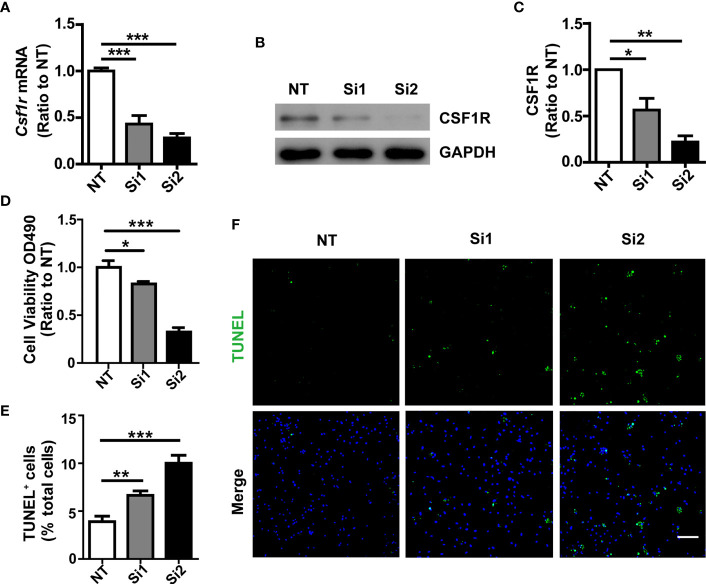
Decreased cell survival in *Csf1r* knockdown microglia. **(A)** *Csf1r* was knocked down in primary microglia by two independent siRNAs and the knockdown efficiency was assessed by qRT-PCR. **(B, C)** *Csf1r* knockdown efficiency was assessed by Western blotting. **(D)** Knockdown of *Csf1r* suppressed microglial viability as examined by MTS assay. **(E, F)** The apoptosis and necrosis of *Csf1r* knockdown microglia were assessed by TUNEL staining. TUNEL-positive cells (green) that co-localized with DAPI were quantified as a percentage of total DAPI-positive cells (blue). The number of TUNEL-positive cells that co-localized with DAPI increased in *Csf1r* knockdown microglia compared with nontarget control (NT). Scale bar, 50 µm. Two-way ANOVA with Tukey’s multiple comparison test. Data are plotted as mean ± SEM (n=3). *p<0.05; **p<0.01; ***p<0.001.

Colony-stimulating factor 1 (CSF1) regulates the survival, proliferation, and differentiation of mononuclear phagocytic cells and is the primary regulator of mononuclear phagocyte production *in vivo* (21). Moreover, genetic loss of the CSF1R blocks the normal population of resident microglia in the brain that originates from the yolk sac during early development. Microglia are >99% depleted at embryonic day 16 and day 1 post-partum brain in mice homozygous for a null mutation in the *Csflr* gene or in mice for selective CSF1R inhibitors extensive treatment (22, 23). To this end, we tested the effects of transitory CSF1R depletion on microglia survival. CSF1R was significantly knocked-down by two independent *siRNAs*, which was verified by qPCR (**Figure 3A**) and Western blotting assay (**Figures 3B, C**). Through genetic manipulation, we found that cell viability was suppressed significantly in *Csf1r* knockdown microglia (**Figure 3D**) as examined by MTS assay. To further quantify the apoptotic and necrotic status of *Csf1r* knockdown microglia by TUNEL staining, we assessed the number of TUNEL-positive cells (green) that co-localized with DAPI compared with total DAPI-positive cells (blue) in those cells (**Figures 3E, F**). The number of TUNEL-positive cells that co-localized with DAPI was significantly increased in *Csf1r* knockdown microglia compared with the NT control microglia (**Figure 3E**). These results suggest that CSF1R is critical for microglia survival.

### CSF1 Activates Akt Signaling in *Trem2*^-/-^ Microglia

**Figure 4:**
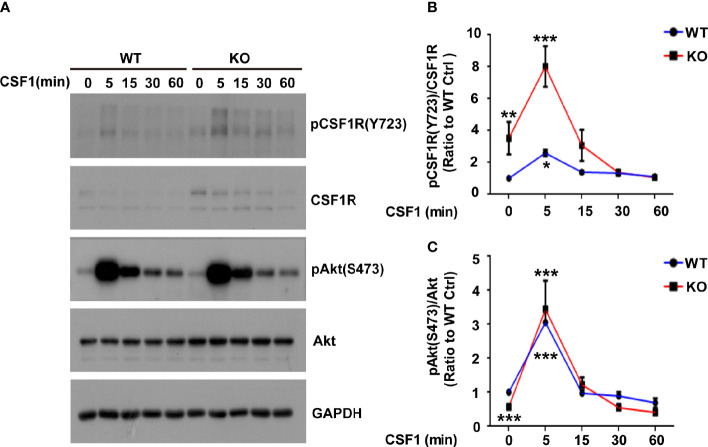
The Akt signal is activated in response to CSF1 in WT and *Trem2* KO primary microglia. **(A)** Primary microglia from WT or *Trem2* KO mice were treated with CSF1 (50 ng/ml) for 5, 15, 30 or 60 mins and cell lysates were analyzed by Western blotting. Representative images of Western blotting for the total and phosphorylation of CSF1R and Akt are shown. **(B)** Protein levels of phosphorylated CSF1R at Tyr723 site (pCSF1R^Tyr723^) were quantified by densitometry and are presented as ratios to GAPDH and total CSF1R. The pCSF1R^Tyr723^ was increased to the highest level at 5-min CSF1 treatment and gradually returned to the normal level. **(C)** Protein levels of phosphorylated Akt (Ser473) were quantified by densitometry and are presented as ratios to GAPDH and total Akt. The pAkt^Ser473^ was increased to the highest level in response to 5-min treatment of CSF1. Two-way ANOVA with Tukey’s multiple comparison test. Data are plotted as mean ± SEM (n=3). Ratio to WT microglia without CSF1 treatment, *p<0.05; **p<0.01; ***p<0.001.

Lack of TREM2 impairs proliferation and β-catenin activation in osteoclast precursors (OcP) in response to CSF1, the ligand of CSF1R (15). Moreover, TREM2 deficiency reduced the viability and proliferation of microglia, and decreased the stability of β-catenin (8). Given that the known ligands for CSF1R are CSF1 and IL34 (24), we then detected these two ligands levels in *Trem2^-/-^* microglia and found that *Csf1* mRNA was not changed whereas *IL-34* mRNA was increased in *Trem2^-/-^* microglia when compared to those in wild-type (WT) microglia (**Supplementary Figures 1A, B**), suggesting that these two ligands of CSF1R may play different role in *Trem2^-/-^* mouse brain. To elucidate whether TREM2 is necessary for CSF1/CSF1R signaling in microglia, *Trem2^-/-^* or WT microglia were cultured in DMEM containing 50 ng/ml CSF1 for 5 min, 15 min, 30 min, and 60 min, respectively. Consistent with previous results in DAP12-deficient macrophages and *Trem2*-deficient OcP (15), the expression of β-catenin in total cell lysates was reduced in the absence of TREM2 and was not restored in *Trem2^-/-^* microglia in response to transient CSF1 treatment (**Supplementary Figures 2A, B**). We then analyzed the phosphorylation of CSF1R and Akt in those cells. The phosphorylation of CSF1R and Akt were elevated both in *Trem2^-/-^* and in WT microglia in response to CSF1 (**Figures 4A–C**), which was also consistent with previous studies that TREM2 was not required for CSF1R-dependent phosphorylation of Akt in OcP cells (15). The increase was highest at 5 min of CSF1 treatment and was more apparent in *Trem2^-/-^* microglia than in WT microglia (**Figures 4A–C**). These results suggest that TREM2 is not necessary for CSF1-mediated CSF1R and Akt phosphorylation in *Trem2*-deficient microglia.

### CSF1 Administration Restores Cellular Survival in *Trem2*^-/-^ Microglia *In Vivo* and *In Vitro*

**Figure 5:**
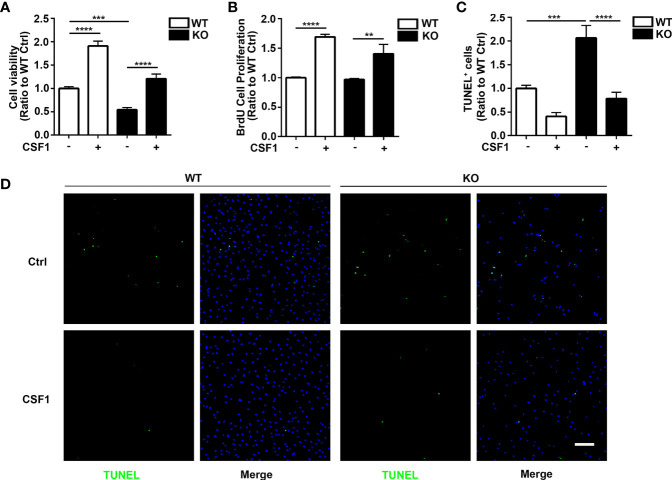
CSF1 restores cell growth and viability in *Trem2* KO microglia. **(A)** Cell viability of *Trem2* KO microglia was enhanced in response to CSF1 treatment using MTS assays. **(B)** CSF1 promotes *Trem2* KO microglia proliferation using BrdU incorporation assay. **(C)** Apoptosis and necrosis of *Trem2* KO microglia were assessed by TUNEL staining. TUNEL-positive, apoptotic cells (green) were quantified as a percentage of total DAPI-positive cells (blue). CSF1 inhibited the apoptosis and necrosis of *Trem2* KO microglia. **(D)** Representative images of TUNEL staining that co-localized with DAPI. Scale bar, 50 µm. Two-way ANOVA with Tukey’s multiple comparison test. Data are plotted as mean ± SEM (n=3). **p<0.01; ***p<0.001; ****p<0.0001.

**Figure 6:**
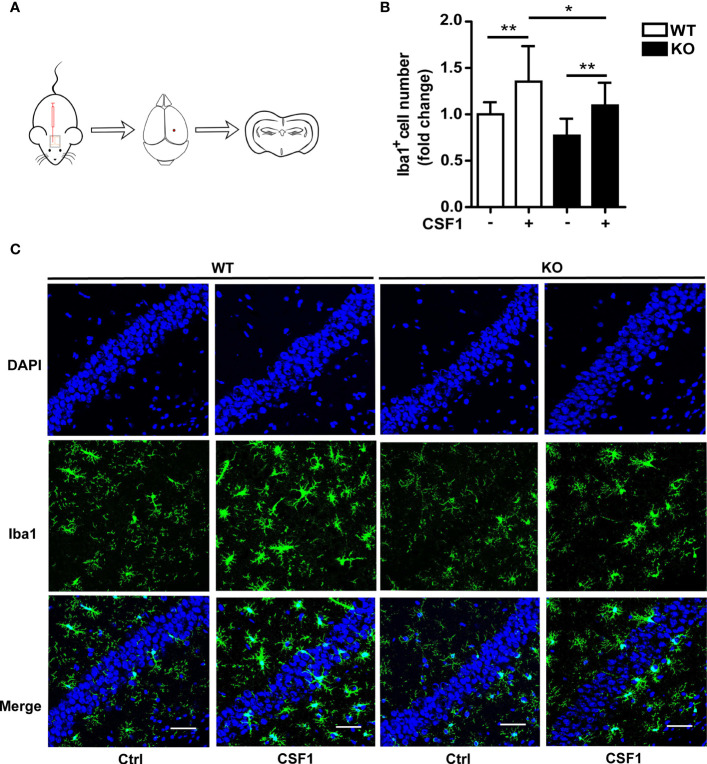
CSF1 administration contributes to microglial survival in *Trem2* KO mouse brain. **(A)** Schematic map of CSF1 intracerebroventricular injection in mouse brain. Two-month-old *Trem2* KO and WT littermates were intralateroventricularly microinjected with 30 ng CSF1 daily for three days. Hippocampi and cortex from the left hemispheres of brains were dissected for protein and RNA extraction, and the right hemispheres of brains were embedded for cryostat sectioning and immunofluorescence. **(B)** Coronal sections from *Trem2* KO or WT mouse brain were stained with Iba-1 (green) for microglia. Number of microglia double stained with DAPI (blue) and Iba1 (green) was quantified per HPF in *Trem2* KO or WT mice. Microglia number response to CSF1 was assessed by quantifying the cell number ratio of CSF1 group to control group in WT or *Trem2* KO brain. **(C)** Representative images of microglial immunofluorescent staining are shown. Two-way ANOVA with Tukey’s multiple comparison test. Data are plotted as mean ± SEM (n=3). *p<0.05; **p<0.01. Scale bar, 50 µm.

Given that CSF1R was increased in *Trem2*^-/-^ microglia and that Akt, an important molecule responsible for maintaining cell survival, was significantly activated in *Trem2^-/-^* microglia in response to CSF1, it is conceivable that CSF1 may have remedial action in microglia survival and microgliosis in *Trem2*^-/-^ microglia and/or in *Trem2*^-/-^ mouse brain. Accordingly, to further evaluate the impact of CSF1 on *Trem2*-deficient microglia survival, *Trem2*^-/-^ or WT microglia were treated with 50 ng/ml CSF1 for 24 hours. As expected, CSF1 promoted the cell viability in *Trem2*^-/-^ microglia as by the MTS assays (**Figure 5A**), although it did not completely restore the effect to the levels in WT cells. Moreover, the proliferation of microglia in *Trem2*^-/-^ microglia was significantly increased in response to CSF1 (**Figure 5B**). Additionally, apoptosis was significantly suppressed in *Trem2*^-/-^ microglia upon CSF1 treatment (**Figures 5C, D**). To evaluate the role of CSF1 in regulating *Trem2*^-/-^ microglial survival *in vivo*, *Trem2*^-/-^ or WT mice were intralateroventricularly microinjected with 30 ng CSF1 daily for three days. We found that CSF1 treatment resulted in a significant increase in the number of microglia in *Trem2*^-/-^ and WT mouse brain though this response in *Trem2*-deficient mice was not the same as in WT mice (**Figures 6A–C**). These results showed that CSF1 treatment partially restored the number of microglia in the *Trem2*^-/-^ mouse brain, indicating that activation of the CSF1R signaling pathway partially restores the survival and/or promotes the proliferation of *Trem2*^-/-^ microglia both *in vitro* and *in vivo*.

### CSF1 Administration Ameliorates Aβ Plaques Accumulation in *Trem2*^-/-^; 5XFAD Mouse Brain

**Figure 7:**
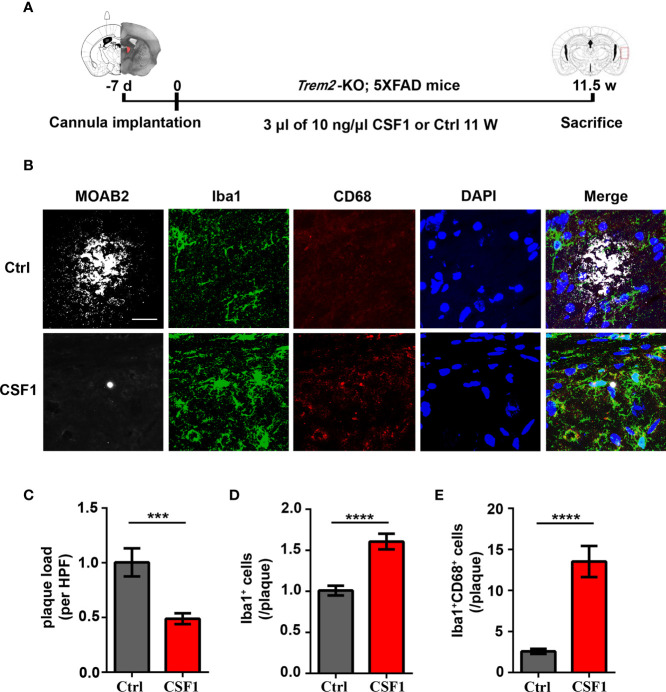
CSF1 administration ameliorates Aβ plaques accumulation in *Trem2*^-/-^; 5XFAD mouse brain. **(A)** Schematic timeline of CSF1 administration in *Trem2*^-/-^; 5XFAD mouse brain. *Trem2*^-/-^;5XFAD mice were treated with 30 ng CSF1 or artificial cerebrospinal fluid-treated controls weekly for 2.5 months. The right hemispheres of the brains were embedded for cryostat sectioning and immunofluorescence. **(B)** Coronal sections of the brains were stained with Iba-1 (green) for microglia, CD68 (red) for phagocytic marker and MOAB2 (gray) for Aβ plaques. Representative images of microglial immunofluorescent staining are shown. **(C)** Immunofluorescence density of Aβ plaques stained with MOAB2 (gray) was quantified per HPF. Aβ plaques immunofluorescence density response to CSF1 was assessed by quantifying the plaques ratio of CSF1 group to control group in *Trem2*^-/-^;5XFAD mouse brain. **(D)** Number of microglia double stained with DAPI (blue) and Iba1 (green) was quantified per HPF. Microglia number response to CSF1 was assessed by quantifying the cell number ratio of CSF1 group to control group in *Trem2*^-/-^;5XFAD mouse brain. **(E)** Number of microglia double stained with CD68 (red) and Iba1 (green) associated with plaques was quantified per HPF. Phagocytic microglia number response to CSF1 was assessed by quantifying the co-staining cell number ratio of CSF1 group to those of control group in *Trem2*^-/-^;5XFAD mouse brain. Student *t* test. Data are plotted as mean ± SEM (n=3). ***p<0.001; ****p<0.0001. Scale bar, 50 µm.

Due to the significant increase in *Trem2*^-/-^ microglial survival in response to CSF1, we next want to know whether CSF1 may play remedial role in Aβ plaques accumulation in *Trem2*^-/-^; 5XFAD mouse brain. Thus, *Trem2*^-/-^; 5XFAD mice were treated with 30 ng CSF1 or ACSF weekly for 2.5 months (**Figure 7A**). Interestingly, MOAB2 staining of plaques revealed that CSF1 treatment resulted in a significant reduction in Aβ plaques accumulation in *Trem2*^-/-^; 5XFAD mouse brain (**Figures 7B, C**). As expected, CSF1 promoted microglial activation in *Trem2*^-/-^; 5XFAD mice as assessed by Iba1 immunofluorescence assays (**Figures 7B, D**). Meanwhile, there was a dramatic enhancement in CD68^+^ Iba1^+^ microglial cells associated with plaques in *Trem2^-/-^*; 5XFAD mouse brain in response to CSF1 treatment, indicating that CSF1 remarkably activated microglia into a phagocytic state (**Figures 7B, E**). These results showed that CSF1 treatment ameliorates Aβ plaques accumulation in the *Trem2*^-/-^; 5XFAD mouse brain, indicating that activation of the CSF1R signaling pathway maybe a therapeutic strategy in AD treatment.

## Discussion

**Figure 8:**
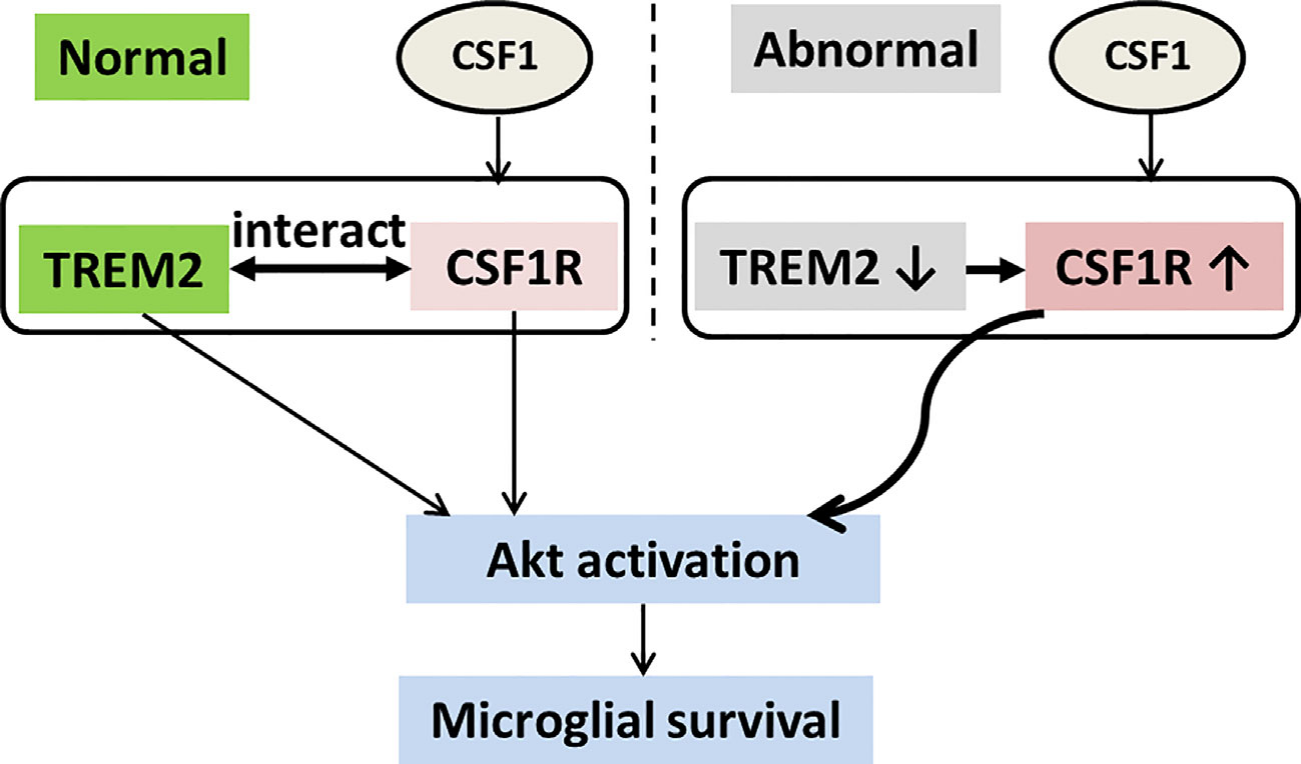
Schematic model of TREM2 and CSF1R interaction in microglial survival. TREM2 and CSF1R interact with each other, both of which promote microglial survival through the Akt pathway. CSF1R expression is increased and has elevated response to CSF1 for Akt activation in *Trem2* deficient microglia.

Herein, we demonstrate the physical association of CSF1R and TREM2 and that TREM2 is not necessary for CSF1/CSF1R signaling mediated microglial survival (**Figure 8**). We further observed that forced knockdown of CSF1R decreased cell survival, which was consistent with previous studies myeloid lineage cells (22, 23). Interestingly, CSF1R was significantly enhanced in *Trem2*^-/-^ microglia and activating CSF1R by CSF1 resulted in significant restoration of the attenuated survival in *Trem2*^-/-^ microglia and in *Trem2*^-/-^ mouse brain due to activation of the Akt signaling pathway (**Figure 8**).

The effects of CSF1 are mediated by the CSF1R tyrosine kinase, and the subsequent phosphorylation of downstream molecules (25). Triggering this phosphorylation cascade increases gene transcription and protein translation, leading to the survival and proliferation of target cells (14). TREM2 is involved in CSF1-mediated survival and proliferation in macrophages and osteoclast precursors. CSF1/CSF1R signals through calmodulin-dependent kinases downstream of DAP12 activates β-catenin in a TREM2-dependent manner (15, 16). Moreover, another study showed that CSF1/CSF1R activated Src kinases, resulting in DAP12 phosphorylation (17). This mechanism suggests that these receptors are in close physical proximity and the functional interaction may potentially influence each other; TREM2 has been proposed as the most obvious candidate receptor responsible for the crosstalk with CSF1R (26). Our study revealed that TREM2 and CSF1R interacted with each other through transmembrane regions. However, the interaction of CSF1R and TREM2 in physiological conditions needs to be further confirmed in future work using practicable TREM2 antibodies. Previously published work revealed an essential role of DAP12 for TREM2 signaling and for CSF1-induced proliferation and survival of macrophages (17), which suggests that the association of DAP12 with a cognate receptor is required (16). As such, whether DAP12 affects the interaction of CSF1R and TREM2 or whether DAP12 is a component of the interaction complex remains elusive. Additionally, given that DAP10 can also interact with DAP12–TREM2 complex and DAP10 was required for the effective activation of phosphoinositide 3-kinase (PI3K) growth factor receptor-bound protein 2 (Grb2), and therefore protein kinase B (Akt) and ERK signaling (27), it may be interesting to investigate whether DAP10 also interacts with the CSF1R-TREM2 complex.

Moreover, it has been proposed that the cytoplasmic domain of CSF1R can translocate to the nucleus (28), but a detailed mechanism for whether the interaction between CSF1R and TREM2 is necessary to target cytoplasmic CSF1R proteins translocated to the nucleus remain elusive. Additionally, recent studies reported that a soluble form of TREM2 (sTREM2) derived from proteolytic cleavage of the cell surface receptor promotes microglial survival in a PI3K/Akt-dependent manner (20), meaning it is desirable to investigate in future whether CSF1R is the receptor that mediates the effects of sTREM2 on microglial survival.

We also found that CSF1R and TREM2 mutually regulate their expression. It is intriguing to note that TREM2 deficiency in microglia led to enhanced CSF1R protein and mRNA level. As expected, knockdown of *Csf1r* in microglia significantly increased *Trem2* mRNA level. These results suggest that CSF1R and TREM2 are complementary in their expression though detail mechanisms need to be clarified. Since both CSF1R and TREM2 are essential in microglial survival, the reactive enhancement of CSF1R in *Trem2* KO microglia may be a salvage measure for microglial survival. Thus, CSF1R and TREM2 have overlapping functions in microglial survival. Emerging evidence suggests that the Akt signaling pathway plays a critical role in cellular survival (29). We have previously reported that Akt was decreased in *Trem2* KO microglia (8), which indicates TREM2 also acts through Akt signaling to mediate microglial survival. Interestingly, the activation of Akt in response to CSF1 in *Trem2*-deficient microglia indicates that CSF1 may be a potential therapeutic molecule for TREM2-loss-of-function neurodegenerative disease. Additionally, Akt inhibitor administration in those experiments can further support our results. Nevertheless, whether the crosstalk of TREM2 with CSF1R signaling and the activation of Akt are involved in the migration of microglia remain to be determined. Furthermore, the similar effects of Akt activation in response to CSF1 in wildtype or *Trem2* KO microglia indicate that TREM2 is not necessary for CSF1/CSF1R/Akt signaling and that there is an unknown signaling pathway of TREM2 in the regulation of microglial survival, which needs to be clarified in future. It is still unclear from our findings whether CSF1R promotes the stabilization of β-catenin and perhaps the subsequent activation of other survival signaling such as Wnt/β-catenin or PI3K/Akt, although previous studies demonstrated that TREM2 signaling *via* its associated adaptor DAP12 synergizes with CSF1R signaling to promote survival of macrophages (15, 16) as well as the survival of primary microglia (8).

Interestingly, the expression of two ligands for CSF1R in *Trem2^-/-^* microglia was different. The increased *IL34* mRNA and unaltered *Csf1* mRNA level in those cells imply their different roles in *Trem2^-/-^* mouse brain. These results also indicate that the increase of IL34 in *Trem2^-/-^* microglia cannot restore microglial viability although the increase needs to be verified by ELISA assay. CSF1 supplements are practical to restore microglial viability in *Trem2^-/-^* mice. Thus one of the most intriguing avenues of this research is that CSF1 can restore cell survival in TREM2 deficient microglia. Since systemic administration of human recombinant CSF1 ameliorates memory deficits in a transgenic mouse model of AD (30, 31), it would not be surprising if future work shows that administration of CSF1 influences this signaling chain in AD therapy.

Our studies also provide direct evidence for the critical role of CSF1R in microglial survival though the molecular mechanisms of how CSF1R deficiency affects the survival of microglia remain elusive. Signaling by CSF1 through CSF1R induced the stabilization and nuclear translocation of beta-catenin, which activated genes involved in the cell cycle. Thus, CSF1R deficiency may result in cell cycle arrest and accelerated apoptosis of microglia. Detailed studies need to be conducted, however, to demonstrate those effects. *Csf1r*^-/-^ mice of most strain backgrounds die perinatally and the surviving mice exhibit multiple developmental deficits with an almost total lack of microglia. In contrast, *Trem2*^-/-^ mice are grossly normal in growth without apparent microglia reduction in young or adult brains but with fewer and abnormal microglia in aged brains (32). Moreover, *Csf1r* haploinsufficiency in humans leads to ALSP, an adult-onset progressive dementia predominantly affecting the cerebral white matter. However, the loss of functions in TREM2 in humans resulted in a severe form of dementia with bone cystic lesions known as Nasu-Hakola disease (NHD), a lethal form of progressive, early onset dementia (33).

The phenotype of the *Csf1r*^-/-^ mouse is more severe than that of the *Trem2*^-/-^ mouse, indicating that CSF1R is more important than TREM2 in mouse growth. Additionally, microglia are reduced by more than 94% in 3-week-old *Csf1r*-null brains (23, 34) and are almost eliminated from the brain upon CSF1R inhibitor treatment (35, 36). Collectively, CSF1R functions directly in microglia survival and the pro-survival effects of TREM2 cannot replace that of CSF1R but only have a synergistic effect with it. These two proteins are required for survival of microglia. While they share most functions, a key difference is that CSF1R modulates microglia survival more efficiently than TREM2. Accordingly, further studies are needed to extend the TREM2 and CSF1R signaling pathway and to identify nuances that are crucial to fully understand their function.

In summary, we show here for the first time that TREM2 interacts with CSF1R and they mutually affect their expression. Importantly, activating CSF1R signaling by CSF1 protects against the phenotypes in *Trem2*-deficient microglia. Nevertheless, there is a potential risk of CSF1R activation in this therapeutic strategy as several studies have shown that CSF1R activation resulted in tumorigenesis (37–39). Thus, the brain-targeting strategies of CSF1R activation need to be seriously considered. Given that augmented β-amyloid (Aβ) accumulates due to a dysfunctional response of microglia in *Trem2*^-/-^; 5XFAD mouse brain, which fail to cluster around Aβ plaques and become apoptotic (40–42), we found CSF1 can ameoliate the augmented Aβ accumulation in *Trem2*-deficient 5XFAD mouse brain by activating microglia into a phagocytic state. Interestingly, studies suggested that CSF1R inhibitors also reduce plaque burden in AD mouse models (35, 43–45). In those studies, microglia influence plaque morphologies during peak pathological progression. The combined data demonstrate a critical role of CSF1R in modulating microglia survival in AD progress. Further studies of behavior tests are required to understand whether CSF1R can be used as a therapeutic target against these diseases.

## Data Availability Statement

The raw data supporting the conclusions of this article will be made available by the authors, without undue reservation.

## Ethics Statement

The animal study was reviewed and approved by Animal Ethics Committee of Xiamen University.

## Author Contributions

HZ and Y-WZ conceived the study and contributed new reagents or analytic tools. BC, XL, and KD performed the research. BC, XH, and XL analyzed the data and interpreted the results. HZ and BC wrote the paper. BC and XL prepared the figures. All other authors discussed and revised the manuscript. All authors contributed to the article and approved the submitted version.

## Funding

This study was supported by grants from the National Natural Science Foundation of China (81771164, 91949129, 81771377, 92049202 and U1705285) and the Open Research Fund of State Key Laboratory of Cellular Stress Biology, Xiamen University (SKLCSB2019KF014). This work was also supported by grant from the Natural Science Foundation of Fujian Province of China (2018D0022 and 2020J01014).

## Conflict of Interest

The authors declare that the research was conducted in the absence of any commercial or financial relationships that could be construed as a potential conflict of interest.
