# Supplementary material for: Triggering Receptor Expressed on Myeloid Cells-2 (TREM2) Interacts With Colony-Stimulating Factor 1 Receptor (CSF1R) but Is Not Necessary for CSF1/CSF1R-Mediated Microglial Survival
Source: Front Immunol. 2021 Mar 25;12:633796. doi: 10.3389/fimmu.2021.633796 (PMC8027073; doi:10.3389/fimmu.2021.633796)
Supplement: Supplementary file 1 [file DataSheet_1.docx]

Supplementary Material

The primer sequences for mouse *Csf1* and *IL34* were as follows: *Csf1*-forward: 5’- GCCAAGGAGGTGTCAGAACA-3’; *Csf1*-reverse: 5’- AGCATTGGGGGTGTTGTCTT-3’; *IL34*-forward: 5’- GGATGTGGAGATTGGCCCTC-3’; *IL34*-reverse: 5’- TACAGCAGTTCCATGACCCG-3’;


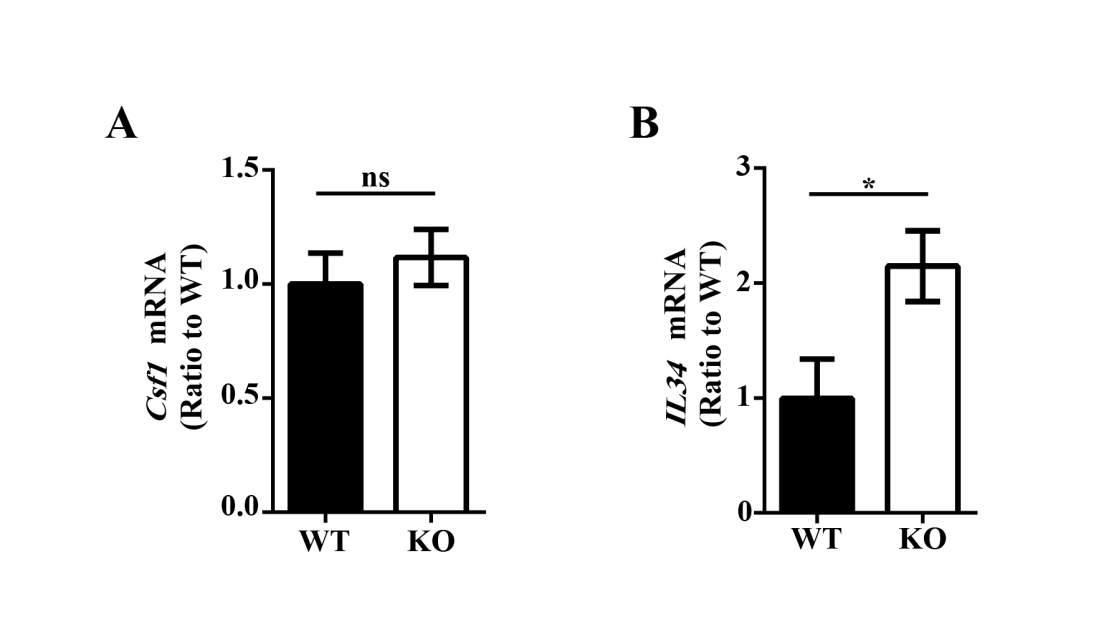


**Supplementary Figure 1.** **The mRNA level of CSF1R ligands in *Trem2* KO microglia. (A)** The mRNA level of *Csf1* was not changed in *Trem2* KO microglia when compared to that in wild-type (WT) microglia. **(B)** The mRNA level of *IL34* was increased in *Trem2* KO microglia when compared to that in WT microglia. Compared to WT control, **p*<0.05.


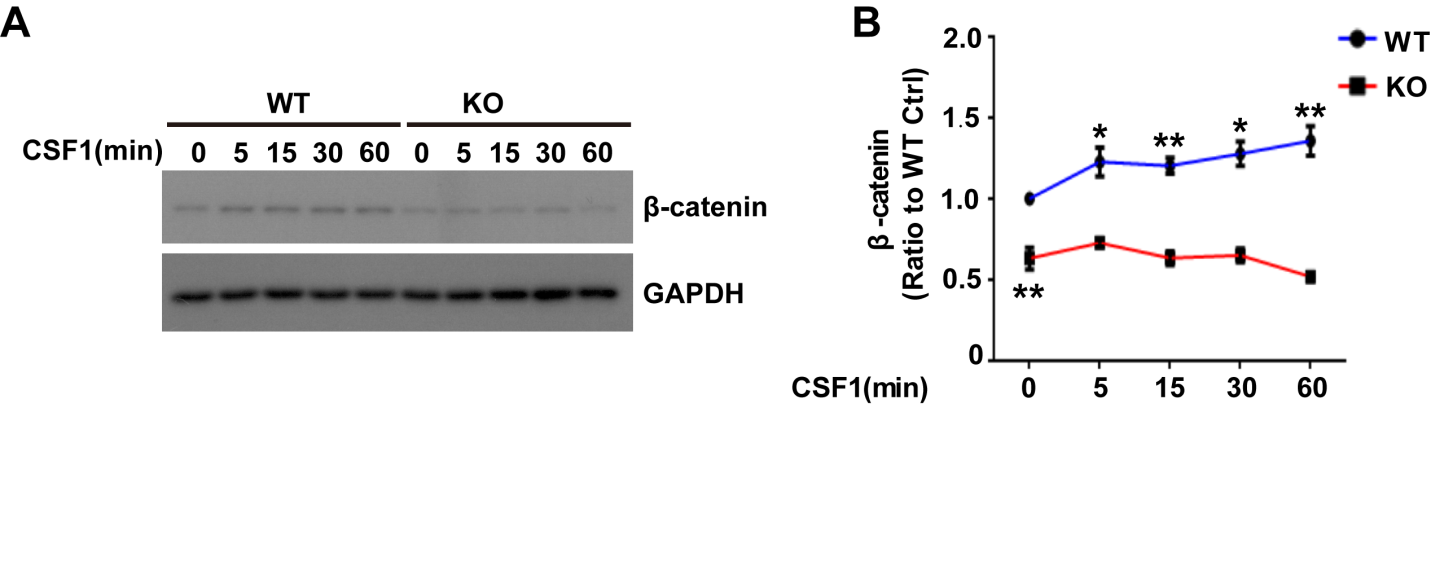


**Supplementary Figure 2.** **TREM2 is necessary for CSF1 mediated β-catenin expression in microglia.**

**(A)** β-catenin protein levels are not changed in response to CSF1 treatment in a short time-dependent manner in *Trem2* KO microglia. **(B)** Representative images of Western blotting for β-catenin protein levels are shown. Compared to WT control, **p*<0.05; ***p*<0.01.
